# Comment on Hosseini et al. Effects of Vitamin D Supplementation on COVID-19 Related Outcomes: A Systematic Review and Meta-Analysis. *Nutrients* 2022, *14*, 2134

**DOI:** 10.3390/nu15010059

**Published:** 2022-12-23

**Authors:** Trudy D. Leong, Ntombifuthi Blose, Denny Mabetha, Tamara Kredo

**Affiliations:** Cochrane-South Africa, South African Medical Research Council, Parow 7503, South Africa

Medicines have been re-purposed as therapeutics for COVID-19 and it is with great interest that we read the publication entitled, “Effects of Vitamin D Supplementation on COVID-19 Related Outcomes: A Systematic Review and Meta-Analysis” [1]. The authors aimed to systematically review the literature to evaluate vitamin D as a therapeutic agent and reported that Vitamin D was associated with a reduced risk of ICU admission and mortality. We note several limitations to the review that were detected when appraising it to inform clinical practice. Notably, data from randomized controlled trials (RCTs) and non-randomized studies of intervention were meta-analyzed with totals of the results of both provided. In addition, there are several concerns regarding an RCT [2] included in the review authored by one of the review authors (BH), however, no declaration regarding potential conflicts of interest was noted. Increasingly, it is recognized that leads of RCTs may have specific views on the intervention under investigation and therefore should declare their interests and potentially recuse themselves from decisions about eligibility, appraisal, analysis, and interpretation of results where their own study is involved [3]. The RCT in question was appraised as low risk of bias across all domains (appraisers BH and HAE). Furthermore, the status of the study is “Terminated” on the clinicaltrials.gov registry (NCT04483635), and “A premature discontinuation was recommended by the Data Safety Monitoring Board and agreed upon by the principal investigator, because the significantly lower recruitment than planned, in the context of mass vaccination of the target population)”-no published study results for the 34 study participants are available for review and we are uncertain how the data was assessed. Authors of an earlier systematic review [4] concluded that evidence for vitamin D supplementation to treat COVID-19 was insufficient and very uncertain and raised concerns regarding substantial clinical and methodological heterogeneity amongst studies (varying supplementation strategies, formulations, vitamin D status of participants, and reported outcomes). This is reflected in decisions by international guidelines groups such as NICE [5], the Pan American Health Organization [6], and Australian National COVID-19 Clinical Evidence Taskforce [6], recommending against the use of vitamin D to treat COVID-19 except as part of a clinical trial. Overall, we suggest that the current knowledge base remains uncertain pending the publication of high-quality RCTs. We, therefore, await the results of ongoing registered studies [7], to inform the evidence base and guide more definitive clinical practice.
